# Crop Phenomics: Current Status and Perspectives

**DOI:** 10.3389/fpls.2019.00714

**Published:** 2019-06-03

**Authors:** Chunjiang Zhao, Ying Zhang, Jianjun Du, Xinyu Guo, Weiliang Wen, Shenghao Gu, Jinglu Wang, Jiangchuan Fan

**Affiliations:** Beijing Key Lab of Digital Plant, Beijing Research Center for Information Technology in Agriculture, Beijing Academy of Agriculture and Forestry Sciences, Beijing, China

**Keywords:** crop phenomics, phenotyping extraction, data storage, functional–structural plant modeling, phenotype–genotype association analysis

## Abstract

Reliable, automatic, multifunctional, and high-throughput phenotypic technologies are increasingly considered important tools for rapid advancement of genetic gain in breeding programs. With the rapid development in high-throughput phenotyping technologies, research in this area is entering a new era called ‘phenomics.’ The crop phenotyping community not only needs to build a multi-domain, multi-level, and multi-scale crop phenotyping big database, but also to research technical systems for phenotypic traits identification and develop bioinformatics technologies for information extraction from the overwhelming amounts of omics data. Here, we provide an overview of crop phenomics research, focusing on two parts, from phenotypic data collection through various sensors to phenomics analysis. Finally, we discussed the challenges and prospective of crop phenomics in order to provide suggestions to develop new methods of mining genes associated with important agronomic traits, and propose new intelligent solutions for precision breeding.

## Introduction

Persistent food and feed supply needs, resources shortages, climate change and energy use are some of the challenges we face in our dependence on plants. Until 2050, crop production will have to double to meet the projected production demands of the global population (Ray et al., 2013). Demand for crop production is expected to grow 2.4% a year, but the average rate of increase in crop yield is only 1.3%. Moreover, production yields have stagnated in up to 40% of land under cereal production (Fischer and Edmeades, 2010). Genetic improvements in crop performance remain the key role in improving crop productivity, but the current rate of improvement cannot meet the needs of sustainability and food security.

Molecular breeding strategies pay more attention to selections based on genotypic information, but phenotypic data are still needed (Jannick et al., 2010; Araus et al., 2014). Phenotyping is necessary to improve the selection efficiency and reproducibility of results in transgenic studies (Gaudin et al., 2013; Yang et al., 2014; Feng et al., 2017) (Table 1). Considering that molecular breeding populations can include a range from at least 200 to at most 10,000 lines, the ability to accurately and high-throughput characterize hundreds of lines at the same time is still challenging (Lorenz et al., 2011; Araus et al., 2014). Apparently, compared with the vast genetic information, there is a phenotyping bottleneck hampering progress in understanding the genetic basis of complex traits. To break this bottleneck and improve the efficiency of molecular breeding, reliable, automatic, and high-throughput phenotypic technologies were urgently needed to provide breeding scientists with new insights in selecting new species to adapt to the resources shortages and global climate change.

**Table 1:** The history of crop phenomics based on major advancement.

The major advancements		References
Germination stage: the Concept formation period of phenotype and phenomics.	The concept of phenotype was first proposed by Danish geneticist Johannsen in 1911.	Johannsen, 2014
	The concept of phenomics corresponding to genomics was first proposed by Nicholas Schork in 1997 in disease research.	Schork, 1997
	Tuberosa proposed the concept that ‘phenotyping was king and heritability was queen’, in 2012.	Tuberosa, 2012
Thriving development stage: from late 20th century, plant phenotypic research teams and commercial organizations were established successively, and a series of high-throughput, high-precision, automated or semi-automated phenotyping tools were developed to obtain high-quality, repeatable plant phenotype data.	In 1998, Belgium CropDesign was the first company to develop a high-throughput phenotyping platform for large-scale plant character evaluation.	http://www.cropdesign.com Reuzeau et al., 2005, 2006; Reuzeau, 2007
	The first research center, named by phenomics-the Australian Plant Phenomics Facility, was established in 2007.	https://www.plantphenomics.org.au
	In 2016, Germany Lemna Tec developed the first field high-throughput plant phenotype platform-Scanalyzer Field, which indicated that plant phenotype technology was formally moving toward the field measurement.	http://www.lemnatec.com Virlet et al., 2016
	Alleviating the micro-phenotyping bottleneck: in recent years, many emerging algorithms and tools have been proposed to handle with microscopic traits of root, stalk and seed, such as RootAnalyzer, VesselParser, etc.	Burton et al., 2012; Du et al., 2016
Systematic development stage: is entering a new era called ’phenomics’, which provides big data and decision support for revealing the molecular mechanism and gene functions of plants.	In 2011, the challenge-phenotyping bottleneck was pointed out by Furbank from the Australian Plant Phenomics Facility, discussing the bottleneck of phenotypic research and the problems need to be solved.	Furbank and Tester, 2011
	The European Plant Phenotyping Network (EPPN) was originated from 2012, which successfully completed the first EPPN joint research project from 2012 to 2015, and continued with the EPPN^2020^ and European Infrastructure for Multi-scale Plant Phenomics and Simulation (EMPHASIS) programs.	https://eppn2020.plant-phenotyping.eu https://emphasis.plant-phenotyping.eu
	In 2013, the concept of next-generation phenotyping was proposed by Mccoueh, suggesting that phenomics should be closely linked to technologies, such as high-resolution linkage mapping, genome-wide association studies and genomic selection models, etc.	Cobb et al., 2013
	The International Plant Phenotyping Network (IPPN) was registered in 2016, representing the world’s major plant phenotyping centers. Over the last decade, a number of national and regional Plant Phenotyping Networks (PPNs) have been organized, such as FPPN. PPA, NAPPN, CPPN, etc., and the communication and cooperation among various PPNs became more and more close.	https://www.plant-phenotyping.org/ Carrolla et al., 2019
	In 2017, Francois Tardieu and Malcolm Bennett presented strategies for multi-scale phenomics. Phenomics research not only needed to build a multi-domain, and multi-scale phenotypic big database, but also to research technical systems for phenotypic traits identification and develop bioinformatics technologies for information extraction from the overwhelming amounts of omics data.	Tardieu et al., 2017

**FIGURE 1:**
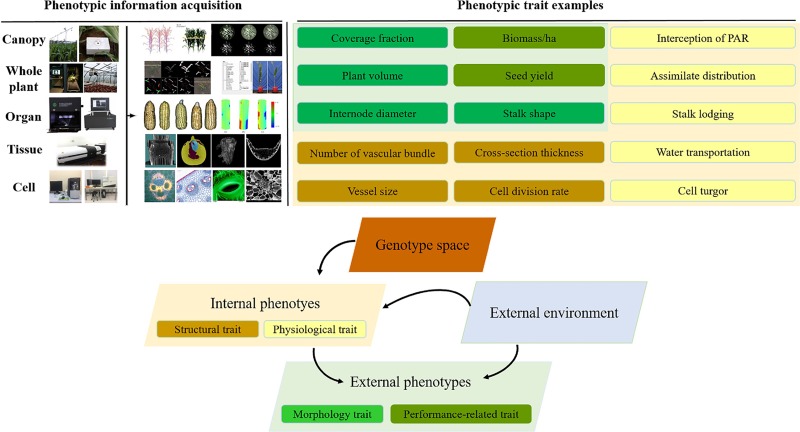
The schematic diagram of genotype–phenotype–envirotype (G-P-E) interactions.

During the past decade, plant phenomics has evolved from an emerging niche to a thriving research field, which was defined as the gathering of multi-dimensional phenotypic data at multiple levels from cell level, organ level, plant level to population level (Houle et al., 2010; Dhondt et al., 2013; Lobos et al., 2017). Crop phenotypes are extremely complicated because they are the result of interaction between genotypes (G) and a multitude of envirotypes (E) (Xu, 2016). This interaction influences not only the growth and development process of crops measured by the structural traits on cellular, tissue, organ and plant level, but also the plant functioning measured by the physiological traits. These internal phenotypes in turn determine crop external phenotypes such as morphology, biomass and yield performance (Houle et al., 2010; Dhondt et al., 2013) (Figure 1). Crop phenomics research integrates agronomy, life sciences, information science, math and engineering sciences, and combines high-performance computing and artificial intelligence technology to explore multifarious phenotypic information of crop growth in a complex environment, of which the ultimate goal is to construct an effective technical system able to phenotype crops in a high-throughput, multi-dimensional, big-data, intelligent and automatically measuring manner, and create a tool comprehensively integrating big data achieved from a multi-modality, multi-scale, phenotypic + environmental + genotypic condition, in order to develop new methods of mining genes associated with important agronomic traits, and propose new intelligent solutions for precision breeding.

Here, we provide an overview of crop phenomics research, focusing on two parts, from phenotypic data collection through various sensors to phenomics analysis. We systematically introduced the crop phenotyping approaches from cellular, tissue, organ, and plant level to field level, discussing application and practical problems in research. Based on overwhelming amounts of phenotypic data, we then discussed the phenotyping extraction, phenotype information analysis and knowledge storage. We emphasize Phenomics is entering the big-data era with multi-domain, multi-level, and multi-scale characteristics. We highlight necessity and importance of building multi-scale, multi-dimensional and trans-regional crop phenotyping big database, researching E-trait depth analysis schemas and technical systems for precise identification of crop phenotypes, realizing functional-structural plant modeling based on phenomics, and developing bioinformatics technologies that integrate genomes and phenotypes.

## Crop Phenotypic Data Collection: Various Phenotyping Approaches for Crop Morphology, Structure, and Physiological Data From Cell to Whole-Plant

Currently, the plant phenotyping community seems some-what divided between high-throughput, low-resolution phenotyping and in-depth phenotyping at lower throughput and higher resolution (Dhondt et al., 2013). Phenotyping systems and tools applied in different scales are focused on different key characteristics –automated phenotyping platforms in controllable environment and high-throughput methodologies in field environment highlights high-throughput, while phenotyping covering the organ, tissue and cellular level emphasizes in-depth phenotyping and higher resolution. In this part, we systematically introduced the crop phenotyping approaches covering from cellular and tissue level to field level, and discussed the application and practical problems of which technologies in crop researches.

### Alleviating the Micro-Phenotyping Bottleneck

Compared with the whole-plant phenotying technologies, phenotyping at higher spatial and temporal resolutions of tissue and cellar scales is more difficult (Hall et al., 2016). To reveal crop micro-phenotypes, the pre-treatment of plant or organ is usually destructive when sampled in a cumbersome and involves multi-step procedure, and the high resolution imaging of samples is also inefficient in view of the output in micron level. More challenging, automated image analysis techniques are urgently needed to quantify cell and tissue traits of crop from the larger high qualified image. In recent years, many emerging algorithms and tools have been proposed to handle with microscopic images from hand-cutting slices, micro-CT, fluorescent, laser, and paraffin sections imaging (Table 2).

Root anatomical traits have important effects on plant function, including acquisition of nutrients and water from the soil and transportation to the aboveground part. Over the last years, novel micro-image acquisition technologies and computer vision have been introduced to improve our understanding of anatomical structure and function of roots. In 2011, computer-aided calculation of wheat root vascular bundle phenotypic information extraction were introduced based on sequence images of paraffin sections (Wu et al., 2011). Burton et al. (2012) (Penn State College of Agricultural Sciences Roots Lab) have developed a high-throughput, high-resolution phenotyping platform, which combines laser optics and serial imaging with three-dimensional (3D) image reconstruction (RootSlice) and quantification to understand root anatomy by semi-auto RootScan (Chimungu et al., 2015). Chopin et al. (2015) developed a fully automated RootAnalyzer software for root microscopic phenotypic information extraction to further improve image segmentation efficiency and ensure high accuracy. Pan et al. (2018) (Beijing Key Laboratory of Digital Plant) introduced X-ray micro-CT into 3D imaging of maize root tissues and developed an image processing scheme for the 3D segmentation of metaxylem vessels. Compared with traditional manual measurement of vascular bundles of maize roots, the proposed protocols significantly improved the efficiency and accuracy of the micron-scale phenotypic quantification.

Different from the root system, crop stalks have a more complex microstructure. The complexity and diversity in microscopic image data poses greater challenges for developing suitable data analysis workflows in the detection and identification of microscopic phenotypes of stalk tissue. Zhang et al. (2013) and Legland et al. (2017) presented semi- automated and automated analysis method for the stained microscopic images of stalk sections. Legland et al. (2014) and Heckwolf et al. (2015) created an image analysis tool that could operate on images of hand-cut stalk transections to measure anatomical features in high throughput. Those tools have significantly improved the measurement efficiency of vascular bundle, but the anatomical traits corresponding to the rind and the detection accuracy remained a challenge. Du et al. (2016) (Beijing Key Laboratory of Digital Plant) introduced micro-computed tomography (CT) technology for stalk imaging and developed the VesselParser 1.0 algorithm, which made it possible to automatically and accurately analyze phenotypic traits of vascular bundles within entire maize stalk cross-sections. So far, Beijing Key Laboratory of Digital Plant has built a novel method to improve the X-ray absorption contrast of maize tissue suitable for ordinary micro-CT scanning. Based on CT images, they introduced a set of image processing workflows for maize root, stalk and leaf to effectively extract microscopic phenotypes of vascular bundles (Zhang et al., 2018).

Phenotyping at tissue and cellar scales still requires complex procedures. The need to simplify the sample preparation process and explore advanced imaging techniques is crucial to accelerate microscopic phenotyping studies. Moreover, image processing is another major bottleneck in micro-phenotyping researches. Due to the specific crop organ and cell phenotypic characteristics of the huge differences, most micro-image analysis algorithms have been developed for specific biological assays currently.

### Three-Dimensional Phenotyping at the Organ Level

Most plant phenotyping platforms concentrated the high-throughput of individual plants (Chaivivatrakul et al., 2014; Cabrera-Bosquet et al., 2016). Hence the phenotypic accuracy of organs on the plants was always compromised (Dhondt et al., 2013). The most frequently used phenotyping index, such as leaf length, leaf area, and fruit volume, could be obtained in phenotyping platforms simply (Klukas et al., 2014; Zhang et al., 2017). The smartphone app platform was developed for field phenotyping by taking pictures and image analysis at organ level (Confalonieri et al., 2017). It is very convenient to acquire leaf angles and leaf length using the app in a 2D view. The plants do not need to be destructively sampled indoors. 2D cameras are low cost and were integrated into most of the phenotyping platforms, which provided effective phenotypic solution for branch structured plants, especially for tracking the dynamic growth of organs on plants (Brichet et al., 2017). However, 2D images lost another dimension data in 3D space, and some of the estimated morphological traits still need to be calibrated (Zhang et al., 2017). Multi-view stereo (MVS) approach (Duan et al., 2016; Vazquez-Arellano et al., 2016; He et al., 2017; Hui et al., 2018) is another popular low-cost alternative for organ level phenotyping. 3D point clouds were reconstructed using multi-view images through structure from motion techniques (Wu, 2011), and then phenotypic traits were extracted through segmented organs of individual plants (Thapa et al., 2018). This cost-effective 3D reconstruction method depicts an alternative to an expensive laser scanner in the studied scenarios with potential for automated procedures (Rose et al., 2015; Yin et al., 2016; Gibbs et al., 2018). There are significant differences between these organ traits within various cultivars, thus tiny errors could be ignored in large scale omics analysis (Yang et al., 2014; Zhang et al., 2017).

**Table 2:** Commonly used and developing approaches for crop micro-phenotypic traits analysis.

Organ type	Image source	Software	Parameters	Remarks	References
Root	Laser Ablation Tomography (LAT)	RootScan RootScan2	Root cross-section, cortex, and stele 3 categories of phenotype indicators	Maize roots	Burton et al., 2012
	Laser dissection microscope	RootAnalyzer	Whole root, tissue regions (cortex, stele, endodermis, metaxylem), stele phenotype indicators	Wheat and maize root	Chopin et al., 2015
	Laser Ablation Tomography (LAT)	RootSlice	Focus on root cortex, including variation in cell size, number of cell files in the radial direction, percentage of aerenchyma, cell wall thickness, amount of cytoplasm and vacuole size.	Maize roots	https://plantscience.psu.edu/research/labs/roots/projects
	Micro-CT images	Simpeware (Commercial software)	Not only the two-dimensional (2D) phenotypic parameters, but also the quantitative analysis of three-dimensional (3D) phenotypic parameters, such as volume and surface area of metaxylem vessels.	Maize roots	Pan et al., 2018
Stalk	Hand-cut stalk transections and color images of which were acquired using a flat scanner	‘Matgeom’, a library for geometric computing with Matlab	Average spatial organization of vascular bundles within maize stalks.	The anatomical traits corresponding to the rind remained a challenge.	http://matgeom.sourceforge.net/
	Hand-cut stalk transections and color images of which were acquired using a flat scanner	The tool, written in the Matlab computer language	Stalk diameter, rind thickness, vascular bundle density, and vascular bundle size.	Maize, sorghum, and Miscanthus stalk. The anatomical traits corresponding to the rind and the detection accuracy of vascular bundle remained a challenge	http://phytomorph.wisc.edu/download/HeckwolfPlantMethods2015/
	Colored with FASGA staining and digitalised with whole microscopy slide scanners	The whole image processing workflow was developed within the ImageJ/Fiji platform	Morphometry (bundle number, rind fraction, etc.) and colorimetry (rind mean red, lignified mean blue, etc.), 2 categories of 19 phenotype indicators	Maize stalk	Zhang et al., 2013
	Micro-CT images	VesselParser 3.0	Stalk diameter, vascular bundle density, vascular bundle size, etc.	This is the first time that quantitative analysis for phenotypic traits of vascular bundles within entire maize stalk cross-section.	Du et al., 2016; Zhang et al., 2018

However, besides these measured length, area, and volume phenotypic parameters, there are lots of obvious differences that can be observed by humans of plant organs, such as blade profiles, blade folds, vein curves, and leaf colors, of which are difficult to obtain the morphological data and quantitatively describe their differences. A series of mathematical approaches (Li et al., 2018) need to be developed to quantitatively describe these differences in order to discover more detailed phenotyping traits with high precision phenotyping data of organ level. Researchers use high resolution 3D scanners to acquire the morphological structure of plant organs (Rist et al., 2018). High resolution 3D scanners are relatively expensive while the acquired morphological data are more accurate than MVS reconstructed organs, especially for non-planner surface plants. 2D LiDAR scanners (Thapa et al., 2018) and depth sensors (Hu et al., 2018) were also used, combined with turn table and translation devices to estimate phenotypic traits through 3D recovery of point clouds. Most 2D or 3D imaging could solve the 3D organ scale phenotyping data acquisition problems, except for tall plants for which it is difficult due to a limited field of vision. High resolution 3D point clouds of plant organs are quite complicated in order to extract more phenotypic traits, thus computer graphics algorithms, such as skeleton extraction (Huang et al., 2013), surface reconstruction (Yin et al., 2016; Gibbs et al., 2017), and feature-preserving remeshing (Wen et al., 2018), need to be applied to process the high-resolution morphological data for 3D phenotypic trait extraction. Because of the difficulty and complexity of detailed organ data acquisition and analysis, Wen et al. (2017) proposed a data acquisition standard and constructed a resource database of plant organs using measured *in situ* morphological data, to realize the integration and sharing of high quality data of plant organs.

**Table 3:** Summary of imaging techniques in high-throughput plant phenotyping platforms (HTPPs).

Imaging technology	Sensors	Raw data	Parameters	Applications
Visible light imaging	Visible light camera	Gray or color value images (RGB channels)	Whole organs or organ parts, time series (minutes to days)	Morphologic traits, digital biomass, height, etc. Assess plant growth status, nutritional status, and accumulated biomass.
Fluorescence imaging	Fluorescence cameras	Pixel-based map of emitted fluorescence in the red and far-red region	Multiple chlorophyll fluorescence parameters and multi-spectral fluorescence parameters	Photosynthetic status/quantum yield/seedling structure/leaf disease, etc.
Infrared imaging	Thermal imaging, Near-infrared cameras	Pixel-based map of surface temperature in the infrared region	Leaf area index, surface temperature, canopy and leaf water status, seed composition, time series (minutes to days)	Measurements of leaf and canopy transpiration, heat dissipation, stomatal conductance differences, etc.
Spectral imaging	Spectrometers, hyperspectral cameras	Continuous or discrete spectra	Water content, seed composition, etc. indoor time series experiment	Disease severity assessment/leaf and canopy growth potential.
3D imaging	Stereo camera/TOF camera systems	RGB/IR/Depth images	Plant or organ morphology, structure, and color parameters, time series at various resolutions	Shoot structure, leaf angle, canopy structure, etc.
Laser scanning	Laser scanning instruments	Depth maps, 3D point clouds	Plant or organ morphology, structure parameters, time series at various resolutions	Shoot structure, leaf angle, canopy structure, etc.
MRI	Magnetic resonance imagers	Water (^1^H) mapping	Water content,, morphology parameters (200–500 μm), 1–600 s	Morphometric parameters/water content.
PET	Positron emission detectors	Radiotracer mapping and co-registration with positron emission signals	Transport partitioning, sectorality, flow velocity, 1–2 mm, 10 s– 20 min	Visualize the metabolic distribution and transport of radionuclides.
CT	X-ray tomography	Voxels/tissue slices	Morphometric parameters in 3D (1–100 μm), minutes- hours	Tissue density, tiller number, seed quality, and tissue 3D reconstruction.

### Automated Phenotyping Platforms in Controllable Environment

Automation and robotics, new sensors, and imaging technologies (hardware and software) have provided an opportunity for high-throughput plant phenotyping platforms (HTPPs) development (Gennaro et al., 2017). In the past 10 years, great improvements have been made in researching and developing HTPPs (Furbank et al., 2011; Fiorani and Schurr, 2013; Virlet et al., 2016). Depending on the overall design, HTPPs in a growth chamber or greenhouse can generally be classified as sensor-to-plant or plant-to-sensor based on whether the plants occupy a fixed position during a measurement routine and an imaging setup moves to each of those positions or the plants are transported to an imaging station, respectively. Collectively, the techniques used in HTPPs in the growth chamber or greenhouse mainly include (Table 3):

(1) RGB imaging, which obtains the phenotypes of plant morphology, color, and texture.
(2) Chlorophyll fluorescence imaging, which obtains photosynthetic phenotypes.
(3) Hyperspectral imaging, which obtains phenotypes such as pigment composition, biochemical composition, nitrogen content, and moisture content.
(4) Thermal imaging, which obtains the plant surface temperature distribution, stomatal conductance and transpiration phenotype.
(5) Lidar, which obtains the three-dimensional structure phenotype of plants.

Moreover, other advanced imaging techniques widely used in medicine, such as MRI, PET, and CT, have also been introduced into HTPPs in the growth chamber or greenhouse. The following table lists the major imaging techniques used in the HTPPs in the growth chamber or greenhouse.

**Table 4:** Exhaustive list of digital technologies and platform equipments for crop phenotyping in controllable environment.

HTPPs		Sensor options	Functions	Application examples	References
WPScan ^Conveyor^		RGB sensor	The world’s first high-throughput phenotyping.	Maize morphologic traits, digital biomass, height, et al.	http://www.wps.eu/en
Trait Mill		RGB sensor	TraitMill is the first platform that combines genes on rice phenotypes.	The TraitMill is a highly versatile tool for testing the effect of genes and gene combinations on plant phenotype. Focusing on japonica rice, but does some work on maize.	http://www.cropdesign.com; Reuzeau, 2007; Reuzeau et al., 2010
Scanalyzer	Plant-to-Sensor	RGB Visible; PS2 Fluorescence; Fluorescence; Near Infrared.	Hosting different sensors to capture multiple data points per plant; Ranging from small versions for few plants up to large installations for several hundreds of plants.	Corn leaf segment; graph to object converter; HSI TO GRAY converter, etc.	Golzarian et al., 2011; Chen D. et al., 2014; Neilson et al., 2015; Amanda et al., 2016; Arend et al., 2016b; Cai et al., 2016; Guo et al., 2017; Liang et al., 2017; Majewsky et al., 2017; Meng et al., 2017; Neumann et al., 2017; Pandey et al., 2017; Parlati et al., 2017; Tomé et al., 2017; van de Velde et al., 2017
	Sensor-to-Plant	Visible light camera; Chlorophyll fluorescence camera; Infrared camera; Hyperspectral cameras; 3D Laser scanner.	Do not to move the plants to avoid mechanical stress; Motorized gantry can be fitted inside the greenhouse to transport sensors above the plants.	Ground cover Canopy height Plant geometry Growth and biomass Counting features Growth stages Vegetation indices Chlorophyll fluorescence parameters	http://www.lemnatec.com
KeyGene digital phenotyping	PhenoFab^®^	RGB sensor	A greenhouse phenotyping platform; The capacity: 1.400 plants.	Seed treatments in sugar beet; Investigate the physiological effects of corn.	http://www.keygene.com
Plant Screen	PlantScreen Modular System	Multiple imaging sensors	Integrated robotic solution for high-precision digital plant phenotyping and plant cultivation of mid-scale size up to large plants in greenhouse or semi-controlled environment.	High-throughput screening Morphology and growth assessment Nutrient management Photosynthetic performance Abiotic stress Pathogen interaction Trait identification Chemical screening Nutrient effects Arabidopsis.	http://www.psi.cz/ Silsbe et al., 2015
PHENOSPEX	PlantEye F500	3D Laser, multispectral camera	Multispectral 3D Scanner for plants	Compute automatically a wide variety of morphological parameters such as: plant height, 3D leaf area, projected leaf area, digital biomass, leaf inclination, leaf area index, light penetration depth and leaf coverage. Typical applications like germination assays, drug screening, experimental control, documentation, quality control or phenotyping.	http://phenospex.com Vadez et al., 2015
	DroughtSpotter	Automated gravimetric sensors	Drought research and breeding		
	MobileDevice	PlantEye F400/PlantEye F500	For lab and greenhouse automation		
Rice automatic phenotyping platform (RAP)		Color imaging device, linear X-ray computed tomography (CT), etc.	A phenotyping facility for high-throughput and automatic phenotypic screening of rice germplasm resources and populations throughout the growth period and after harvest.	Combination of the multifunctional phenotyping tools RAP and GWAS to investigate the genetic control of rice and maize growth and development.	http://plantphenomics.hzau.edu.cn/; Yang et al., 2015; Zhang et al., 2017
SCREEN House	PlantScreen Self-Contained (SC) Systems/ PlantScreen Compact System	RGB digital color imaging, Kinetic, chlorophyll fluorescence Imaging, Hyperspectral Imaging, Thermal Imaging, 3D Scanning, etc.	The system is designed for digital phenotyping of small and mid-sized plants up to 50 cm in height.	Arabidopsis, strawberries, turf grass, soybean, tobacco, corn seedlings, etc. Morphology and growth assessment Nutrient management Photosynthetic performance Abiotic stress Pathogen interaction Trait identification Chemical screening Nutrient effects	http://qubitphenomics.com Berger et al., 2007
SCREEN House		RGB camera	Monitoring plant water status; shoot structure of plant.	This system is used for screening of the shoot structure and function of different plant species (e.g., canola, maize, tomato, cereals) in a greenhouse; Continuous monitoring of plant water status and the environmental conditions	Nakhforoosh et al., 2016
PHENOPSIS		RGB camera, infrared camera.	Automated phenotyping platform allowing a culture of approximately 200–500 Arabidopsis plants in individual pots with automatic watering and imaging system	Rosette area or leaf area measurements through image analysis; Photosynthesis and stomatal conductance measurements.	http://bioweb.supagro.inra.fr/phenopsis Granier et al., 2006

After integration of information technology, digital technology and platform equipment with plant phenotyping, several HTPPs in growth chamber or greenhouse have been listed in Table 4. Such high-throughput phenotyping platforms are characterized by automation, high-throughput, and high precision, which greatly improve plant data collection efficiency and accuracy, in order to improve the efficiency of crop breeding.

However, most HTPPs still have high construction, operating, and maintenance costs, and most academic and research institutions do not have access to these techniques as a result (Kolukisaoglu and Thurow, 2010). For example, the number of fully automatic and high-throughput phenotyping platforms is limited in China: one was developed by the Crop Phenotyping Center (CPC), http://plantphenomics.hzau.edu.cn/, and the others were introduced by LemnaTec, such as http://bri.caas.net.cn/, http://www.kenfeng.com/, and http://www.zealquest.com/. Because of this, there has been increased research into developing affordable, small-scale plant phenotyping platforms and technology. In fact, affordable phenotypic acquisition techniques or platforms are constantly being updated, such as the imaging system developed by Tsaftaris and Noutsos (2009) that uses wireless-connected consumer digital cameras, and the low-cost Glyph phenotyping platform (Pereyra-Irujo et al., 2012). Lowering the cost of these platforms could, therefore, significantly increase the scope of phenotypic research and advance the rapid expansion of phenotypic–genotypes analysis for complex traits.

### High-Throughput Methodologies for Crop Phenotyping in Field Environment

Field-based phenotyping (FBP) is a critical component of crop improvement through genetics, as it is the ultimate expression of the relative effects of genetic factors, environmental factors, and their interaction on critical production traits, such as yield potential and tolerance to abiotic/biotic stresses (Araus and Cairns, 2014; Neilson et al., 2015). Currently, the most commonly field-based phenotyping platforms (FBPPs) use ground wheeled, rigid motorized gantry or aerial vehicles, combined with a wide range of cameras, sensors and high-performance computing, to capture deep phenotyping data in time (throughout the crop cycle) and space (at the canopy level) in field environments (Fritsche-Neto and Borém, 2015) (Table 5). It cannot be denied that the efficiency of ground wheeled phenotyping system is quite low if the plot area is too large (White et al., 2012; Zhang and Kovacs, 2012). In 2016, FIELD SCANALYZERS, with rigid motorized gantry supporting a weather proof measuring platform that incorporates a wide range of cameras, sensors and illumination systems, were developed. The facility, equipped with high-resolution visible, chlorophyll fluorescence and thermal infrared camera, hyperspectral imager, and 3D laser scanner. Crops within a 10–20 m × 110–200 m area can be monitored, which realizes the continuous, automatic, and high-throughput detection of crop phenotyping detection in field (Virlet et al., 2016; Sadeghi-Tehran et al., 2017). Meanwhile, the cable-suspended field phenotyping platform covering an area of ∼1 ha was also developed for rapid and non-destructive monitoring of crop traits (Kirchgessner et al., 2016). However, these large ground-based field phenotyping platforms also have high construction, operating, and maintenance costs, and it has to be located at certain sites limiting the scale at which it can be used. Furthermore, the ground-based FBPPs are not very suitable for large crops, such as maize, except in the early stages (Montes et al., 2011).

In recent years, manned aircraft and unmanned aerial vehicle remote sensing platforms (UAV-RSPs) are becoming a high-throughput tool for crop phenotyping in the field environment (Berni et al., 2009; Liebisch et al., 2015), which meet the demands of spatial, spectral, and temporal resolutions (Yang et al., 2017) (Table 5). For example, thermal sensors fitted to manned aircraft were used to measure canopy temperature (Deery et al., 2016; Rutkoski et al., 2016). The sensors that UAV-RSPs carried typically included digital cameras, infrared thermal imagers, light detection and ranging (LIDAR), multispectral cameras, and hyperspectral sensors, which are applied to: canopy surface modeling and crop biomass estimation based on visible imaging; crop physiological status monitor, such as chlorophyll fluorescence and N levels, based on visible–near-infrared spectroscopy and high-resolution hyperspectral imaging; plant water status detection based on thermal imaging; and crop fine-scale geometric traits analysis based on LIDAR point clouds (Sugiura et al., 2005; Overgaard et al., 2010; Swain et al., 2010; Wallace et al., 2012; Gonzalez-Dugo et al.; Gonzalez-Dugo et al., 2014; Mathews and Jensen, 2013; Diaz-Varela et al., 2014; Gonzalez-Dugo et al., 2015; Nigon et al., 2015; Gómez-Candón et al., 2016; Camino et al., 2018; Roitsch et al., 2019). There are definite advantages for UAV-RSPs, including portable, high monitoring efficiency, low-cost, and suitability for field environments. On the other hand, some limiting factors for UAV-RSPs also exist, including the lack of methods for fast and automatic data processing and modeling, the strict airspace regulations, and vulnerable to different weather conditions. Recently, combining ground-based platforms and aerial platforms for phenotyping offers flexibility. For example, the tractor-based proximal crop-sensing platform, combined with UAV-based platform, was used to target complex traits such as growth and RUE in sorghum (Potgieter et al., 2018; Furbank et al., 2019).

**Table 5:** Exhaustive list of characteristics and application of field phenotypic platforms.

Field-based phenotyping platforms (FBPPs)		Sensor options	Functions	Application examples	References
Ground-based field phenotyping platforms	Field Scanalyzers	Visible light; Infrared imaging; Hyperspectral imaging; PS2 Fluorescence; Laser Scanners; environmental sensors.	Capture deep phenotyping data from crops and other plants growing in field environments.	Ground cover, Canopy height, Plant geometry, Growth and biomass, Counting features, Growth stages, Vegetation indices, Chlorophyll fluorescence parameters.	Virlet et al., 2016; Sadeghi-Tehran et al., 2017
	FieldScan	PlantEye sensors	For ultra-high-throughput plant phenotyping under field- or semi-field conditions with throughputs of 5,000 plants or higher per hour.	Automatically compute a wide variety of morphological parameters such as: Plant height, 3D leaf area, Projected leaf area, Digital biomass, Leaf inclination, Leaf area index, Light penetration depth, Leaf coverage.	http://phenospex.com Vadez et al., 2015
	PlantScreen Field Systems	Hyperspectral imaging; Fluorescent imaging; Thermal imaging	An autonomous drive pivot tower contains multiple sensor nodes mounted on an XZ– robotic arm.	Plant height evaluation and leaf overlap detection, rapid non-invasive measurement of photosystem II activity, analysis of plant’s responses to heat load and water deprivation, and 3D plant reconstruction	http://qubitphenomics.com
	ETH Field Phenotyping Platform (FIP)	DSLR; laser scanner; thermal camera.	Cable-suspended field phenotyping platform covering an area of ∼1 ha	Monitoring canopy cover, canopy height and traits related to thermal and multi-spectral imaging of selected examples from winter wheat, maize and soybean.	Kirchgessner et al., 2016
	Phenomobile Lite	LiDAR; RGB camera; hyperspectral camera; thermal camera.	A variety of crops less than 1.5 m in height. Can be adapted for row/vine crops	Non-destructive field phenotyping of both wheat and rice yielding estimates of canopy height, fractional ground cover, greenness vertical distribution, leaf area, plant counts, visual assessments.	https://www.plantphenomics.org.au/ Jimenez-Berni et al., 2018
UAV platform	Airborn	LiDAR; Hyperspectral camera	Plant height estimation, LAI estimation, Biomass estimation, Leaf N concentration detection	Maize and wheat Plant height, LAI, aboveground Biomass; Potato leaf N concentration	Li et al., 2014, 2015; Nigon et al., 2015; Tattaris et al., 2016
	Multi-rotor UAV	RGB camera; multispectral camera; hyperspectral camera; thermal camera.	Physiological conditions assessment, crop growth monitoring, green canopy cover and LAI estimation, Plant height and biomass estimation, Vegetation monitoring.	Barley, soybean, maize, sunflower, wheat, rice, onion, citrus, vineyard phenotypic analysis.	Zarco-Tejada et al., 2014; Yang et al., 2017; etc.
	Fixed-wing UAV	RGB camera; multispectral camera; hyperspectral camera; thermal camera.	Lodging estimation, weed detection, estimation of net photosynthesis, grain yield prediction, stress detection.	Maize, citrus, vineyard, peach phenotypic analysis.	Overgaard et al., 2010; Li et al., 2014; Yang et al., 2017
	Flying wing	Multispectral camera	Agricultural surveillance and decision support.	Cherries mature ratio.	Herwitz et al., 2004
	Helicopter	RGB camera; multispectral camera.	Ground cover estimation; yield prediction, biomass estimation, LAI and Chlorophyll estimation.	Sorghum, rice, corn, olive phenotyping detection.	Berni et al., 2009; Swain et al., 2010; Chapman et al., 2014

## Crop Phenomics: From Phenotyping Extraction, Data Storage to Knowledge Analysis

Phenomic experiments are not directly reproducible because of the multi-model of sensors (structure, morphology, color, and physiology information), multi-scales phenotypic data (from cellular to population level), and variability of environmental conditions. Crop phenotypic data collection is only the first step in Phenomic research. How to extract phenotypic traits from raw data? How to realize phenotype data standardization and storage? How to make cross-scale, cross-dimensionality meta-analyses? Finally, based on the phenotypic big data, how to realize the model-assisted phenotyping and phenotypic-genomic selection? Hereby this part focuses on hot topics in crop phenomics analysis raised above. We then suggest that research in this area is entering a new stage of development using artificial intelligence technology, such as deep learning methods, in which can help researchers transform large numbers of omic-data into knowledge.

### Phenotype Extraction

Image-based phenotyping, as an important and all-purpose technique, has been applied to measure and quantify vision-based and model-based traits of plant in the laboratory, greenhouse and field environments. So far, lots of image analysis methods and softwares have been developed to perform image-based plant phenotyping (Fahlgren et al., 2015b). From image analysis perspective, phenotypic traits of plant can be classified into 4 categories, i.e., quantity, geometry, color and texture, and are also classified into linear and non-linear features related to the pixel representation. Moreover, some valuable agronomic and physiological traits can be derived from image features. Generally, image techniques are specially designed according to specific crop varieties and phenotypic traits of interest, and always require with prior knowledge of research objects, as well as more or less man-machine interaction. Under highly controlled conditions, the classic image processing pipeline can provide acceptable phenotyping results, such as biomass (Leister et al., 1999), NDVI (Walter et al., 2015), chlorophyll responses (Walter et al., 2015), and compactness (Vylder et al., 2012) etc. However, the simple image processing pipeline is still very difficult to handle with non-linear, non-geometric phenotyping tasks (Pape and Klukas, 2015). Facing species and environment diversities, fully automated and intelligent image analysis will remain a long-term challenge.

Machine learning techniques, such as support vector machines, clustering algorithms, and neural networks, have been widely utilized to image-based phenotyping, which not only improve the robustness of image analysis tasks, also relieve tedious manual intervention (Singh et al., 2016). There is no doubt that machine learning techniques will have a prominent role in breaking through the bottlenecks of plant phenotyping (Tsaftaris et al., 2016). In a broad category of machine learning techniques (Ubbens and Stavness, 2017), deep learning demonstrates impressive advantages in many image-based tasks, such as object detection and localization, semantic segmentation, image classification, and others (Lecun et al., 2015). In essence, deep convolutional neural networks (CNNs) are well suited to many vision-based computer problems, e.g., recognition (Lecun, 1990), classification (He et al., 2015), and instance detection and segmentation (Girshick, 2015). Compared with the traditional image analysis methods, CNNs are simultaneously trained from end to end without image feature description and extraction procedures. As far as plant phenotyping, CNNs have been effectively applied to detect and diagnosis (Mohanty et al., 2016), classify fruits and flowers (Pawara et al., 2017), and count leaf number (Ubbens and Stavness, 2017). It is worth noting that those vision-based phenotyping tasks were driven by the massive captured and annotation plant images. From the view of machine vision perspective, deep learning has been a fundamental technique framework in image-based plant phenotyping (Tsaftaris et al., 2016).

### Phenotype Data Standardization and Storage

A huge amount of complex data and the integration of a wide range of image, spectral and environmental data can be generated through by the above phenotypic technologies, usually up to GB or even petabytes, unstructured “Big Data.” Thus, the efficient storage, management and retrieval of phenotypic data have become the important issues to be considered (Wilkinson et al., 2016). The current universally accepted principle of information standardization includes three aspects: (i) the ‘minimum information’ (MI) approach is recommended to define the content of the data set, (ii) ontology terms is applied for the unique and repeatable annotation of data, and in the form of data sharing and meta-analyses (Krajewski et al., 2015; Coppens et al., 2017), (iii) and proper data formats, such as CSV, XML, RDF, MAGE-TAB, etc., are chosen for the construction of data sets. Up until now, a number of phenotyping resources have been built ranging from phenotypic data of one species to multi-data types (Coppens et al., 2017). In 2010, PODD was developed for capturing, managing, annotating and distributing the data to support both Australian and international biological research communities (Li et al., 2010); Fabre et al. (2011) built a PHENOPSIS DB information system for *Arabidopsis thaliana* phenotypic data acquired by the PHENOPSIS phenotyping platform; Bisque is the first web based, cross-platform, developed into a repository to store, visualize, organize and analyze images in the cloud (Kvilekval Das et al., 2010; Goff et al., 2011). In 2014, ClearedLeaves DB, an on open online database, was built to store, manage and access leaf images and phenotypic data (Das et al., 2014); AraPheno^1^ was the first comprehensive, central repository of population-scale phenotypes (it integrated more than 250 publicly available phenotypes from six independent studies) for *A. thaliana* inbred lines (Seren et al., 2017); PhenoFront was a publicly available dataset of above-ground plant tissue to the LemnaTec Phenotyper platform (Fahlgren et al., 2015a); in 2016, the plant genomics and phenomics research data repository (PGP) were developed by the Leibniz institute of plant genetics and crop plant research and the German plant phenotyping network to comprehensively publish plant phenotypic and genotypic data (Arend et al., 2016a). Obviously, from the perspective of database data standardization and storage, the storage scheme based on “cloud technology” is becoming the trend for the development of plant phenotype data storage. Cloud storage system can optimize the design of the system architecture, file structure, high-speed cache, etc., for the plant phenotype platform. At present, all kinds of phenotypic data collection platforms are still relatively independent, and have not been established at the level of regions, countries or continents. Through the advanced technology of artificial intelligence, establishing a typical crop phenotype database based on the multi-layer phenotypic information, for example GDB Human Genome Database, will of interest to a range of stakeholders.

### Model-Assisted Phenotyping: Functional–Structural Plant Modeling

Plants are highly plastic to genotypes, environment and management via changing morphological traits and adjusting their physiological behavior. Complex interactions between genotypes, environment and managements at different scales determine the development of plants, but their separate contribution to the phenotype remains unclear. Dynamic models have been proven to be an efficient tool in dissection of abiotic and biotic effects on plant phenotypes (Tardieu and Tuberosa, 2010). Functional–structural plant (FSP) (Vos et al., 2010) models simulate plant growth and development in time and three-dimension (3D) space, and quantify complex interactions between architecture and physiological processes. The combination of FSP modeling and phenotyping have been used as a facile technique to address research questions in two ways.

Firstly, FSP models offer a tool to dissect phenotype governed by a set of mechanisms. For example, Zhu et al. (2015) used a FSP model to dissect net biodiversity effect into the effect induced by interspecific trait differences, and the effect induced by phenotypic plasticity by simulating whole-vegetation light capture for scenarios with and without phenotypic plasticity based on experimental plant trait data. The separate effect of each architectural trait (leaf angle, leaf curvature and internode length etc.) on dry mass production and light interception was quantified by simulating canopy growth using a dynamic FSP model for tomato (Chen T.W. et al., 2014). Radiation interception and radiation use efficiency were dissected into an environmental and a genetic term via conducting virtual multi-genotype canopies, in which the FSP model for maize was applied to calculate light interception for each plant (Chen et al., 2018). FSP modeling has proven to be highly effective for disentangling the relative contribution of each underlying process.

Secondly, high-throughput phenotyping techniques facilitate the automate and precise calibration of FSP models. Image-based analysis was performed daily to reconstruct individual maize architecture, in order to calculate light interception using a FSP model and to estimate leaf area and the fresh plant weight of individual plants (Cabrera-Bosquet et al., 2016). Terrestrial LiDAR scanning was used to reconstruct complex tree canopy for predicting three-dimensional distribution of microclimate-related quantities in terms of net radiation, surface temperature and evapotranspiration (Bailey et al., 2016). OPENSIMROOT integrated a root model that can simulate growth of a root system with 3D phenotyping techniques, such as magnetic resonance imaging (MRI) and X-ray computed tomography (CT) (Postma et al., 2017). Phenotyping techniques not only provide an efficient method to evaluate the ability of the model to simulate plant architecture and geometry but also help researchers to understand functional responses based on images.

### Phenotype–Genotype Association Analysis

Although genomic data has a major role in crop genetic improvements and breeding programs, with the advent of the era of omics, considerable gain can only be achieved by tightly coupling genomic discovery to plant phenomics (Cobb et al., 2013; Bolger et al., 2017). In recent years, phenomic researches that combine genomic data with data on quantitative variation in phenotypes have been initiated in many species, which rapidly decoded the function of a mass of unknown genes and improved understanding the G-P map (Campbell et al., 2015, 2017; Neumann et al., 2017).

Many agronomic traits are complex and controlled by many genes, each with a small effect. Identifying the molecular basis of such complex traits requires genotyping and phenotyping of suitable mapping populations, enabling quantitative trait locus (QTL) mapping and genome-wide association studies (GWAS), which have been widely carried out in crop plants (Salvi and Tuberosa, 2015; Muraya et al., 2017). Busemeyer et al. (2013) associated phenotypic traits of small grain cereals with genome information to dissect the genetic architecture of biomass accumulation. In 2014, based on 13 traditional agronomic traits and 2 newly defined traits of rice, Yang et al. (2014) identified 141 associated loci by GWAS. In 2015, Combining GWAS with 29 leaf traits at three growth stages using high-throughput leaf scoring (HLS), 73 new loci with leaf size, 123 of leaf color, and 177 of leaf shape were detected (Yang et al., 2015). In 2017, large-scale quantitative trait locus (QTL) mapping was performed, combined with 106 agronomic traits of maize inbred line from seedling to tasselling stage, and a total of 988 QTLs were identified (Zhang et al., 2017). Also, plant sizes of 252 diverse maize inbred lines were monitored at 11 different developmental time points, and 12 main-effect marker-trait associations were identified (Muraya et al., 2017).

Obviously, combining the high-throughput phenotyping technology and large-scale QTL or GWAS analysis not only greatly expanded our knowledge of the crop dynamic development process but also provided a novel tool for studies of crop genomics, gene characterization and breeding. We believe that with a complete system of genetic information, combined with crop high-throughput phenotyping technology, phenotypic-genomic analysis will revolutionize how we deal with complex traits and underpin a new era of crop improvement.

## Future Challenges and Prospects

Phenomics is entering the era of ‘Big Data,’ thus the crop science community need to combine artificial intelligence technology and collaborative research at the national and international levels, to build a new theory for analyzing crop phenotypic information, construct an effective technical system able to phenotype crops in a high-throughput, multi-dimensional, big-data, intelligent and automatically measuring manner, and create a tool comprehensively integrating big data achieved from a multi-modality, multi-scale, phenotypic + environmental + genotypic condition. There is no denying that there are many challenges that crop phenomics need to address in the next 5–10 years, such as:

(1) Phenomics is entering the big-data era with high-throughput, multi-dimensionality, and multi-scale. We emphasize various phenotyping approaches for crop morphology, structure, and physiological data with three multi- characteristics: multi-domain (phenomics, genomics etc.), multi-level (traditional small to medium scale up to large-scale omics), and multi-scale (crop morphology, structure, and physiological data from cell to whole-plant). The single and individual phenotypic information cannot satisfy the association analysis in the new era called ‘-omics,’ and the systematic and complete phenomics information will be the foundation of future research.
(2) In response to emerging challenges, new methods and techniques based on artificial intelligence shall be introduced to advance image-based phenotyping. An automated phenotyping system and platform result in lots of digital features, which need to prove their values throughout large sample statistics and relationship analysis with traditional agronomic traits. How to precisely and efficiently evaluate, understand and interpret these digital image-based features, and dig out valuable quantitative traits for functional genomes are key problems in the development and application of plant phenotyping.
(3) With the multi-domain, multi-level, and multi-scale phenotypic information, we urgently need to make use of the latest achievements of artificial intelligence in depth learning, data fusion, hybrid intelligence and swarm intelligence to develop big-data management producers for supporting data integration, interoperability, ontologies, shareability and globality.
(4) Modeling is a powerful tool to understand G × E × M interactions, identify key traits of interest for target environments. Nevertheless, several scientific and technical challenges need to be overcome. For example the validity and practicality of the models in terms of modeling processes and their interactions need further verification, and the interaction and feedbacks of multi-scale phenotypes between modeling processes also need to be solved. Only then will we be able to streamline and speed up the tortuous gene-to-phenotype journey through modeling to develop the required agricultural outputs and sustainable environments for everybody.
(5) Crop genotype (G) -phenotype (p) -envirotype (E) information comprehensive analysis and utilization. In short, as Coppens et al. (2017) said “the future of plant phenotyping lies in synergism at the national and international levels.” We need to seek novel solutions to the grand challenges of multi-omics data, such as intelligent data-mining analytics, which offers a powerful tool to unravel the biological processes governing plant growth and development, and to advance plant breeding for much-needed climate-resilient and high-yielding crops.

## Author Contributions

CZ, XG, and YZ conducted the literature survey and drafted the article. JD, WW, SG, JW, and JF provided the data and tables about the crop phenotyping researches by NERCITA. All authors read and approved the final manuscript.

## Conflict of Interest Statement

The authors declare that the research was conducted in the absence of any commercial or financial relationships that could be construed as a potential conflict of interest.

## Footnotes

1 https://arapheno.1001genomes.org
